# Mounier‐Kuhn Syndrome Flare With Pleuritic Chest Pain: A Discussion of Complications and Management

**DOI:** 10.1155/crpu/7654438

**Published:** 2026-02-27

**Authors:** Geran Maule, Akil Augustus, Mohammad Khraisat, Jay Mehta, Elio Rodríguez Araque, Michael D. Schweitzer

**Affiliations:** ^1^ Department of Clinical Sciences, University of Central Florida College of Medicine, Orlando, Florida, USA, ucf.edu; ^2^ Department of Graduate Medical Education, HCA Florida North Florida Hospital, Gainesville, Florida, USA; ^3^ Department of Medicine, Milton Cato Memorial Hospital, Kingstown, Saint Vincent and the Grenadines; ^4^ Department of Medicine, Universidad Central de Venezuela, Caracas, Venezuela, ucv.ve; ^5^ Department of Pulmonary and Critical Care Medicine, University of Florida College of Medicine, Jacksonville, Florida, USA, ufl.edu

## Abstract

**Background:**

Mounier‐Kuhn syndrome (MKS), or tracheobronchomegaly, is a rare disorder characterized by dilation of the trachea and main bronchi due to atrophy of elastic and smooth muscle fibers. It is associated with recurrent respiratory infections, bronchiectasis, and airway collapse. Although radiological diagnosis is well established, clinical recognition remains delayed in many cases due to the disease′s rarity and nonspecific presentation.

**Case Presentation:**

We present a 56‐year‐old male with a known history of MKS who presented with pleuritic chest pain, productive cough, and constitutional symptoms. Computed tomography (CT) scans of the chest showed significant tracheobronchomegaly with posterior tracheal diverticulum and cystic bronchiectasis. Laboratory evaluation was unremarkable, and infectious and autoimmune workups were negative. The patient was treated conservatively with pulmonary hygiene, bronchodilators, and symptom‐targeted therapy, leading to gradual improvement.

**Discussion:**

This case dives into a noninfectious symptomatic flare of MKS, expanding the clinical understanding of its complications. We explore the pathophysiology of pleuritic pain in this setting and review diagnostic delays, bronchiectasis‐related morbidity, and therapeutic strategies. Advances in interventional pulmonology, including airway stenting and tracheobronchoplasty, offer promising outcomes in selected patients. Early identification, structured imaging assessment, and a multidisciplinary approach are key in managing this complex syndrome.

**Key Learning Points**

• Mounier‐Kuhn syndrome (MKS) can present with noninfectious pleuritic pain, complicating diagnostic evaluation.
• Supportive therapy remains the mainstay in mild flares.
• Radiologic features such as tracheal diverticula and cystic bronchiectasis should prompt consideration of MKS in undiagnosed patients with chronic respiratory symptoms.

## 1. Introduction

MKS, also known as congenital tracheobronchomegaly, is a rare structural disorder of the central airways characterized by abnormal dilatation of the trachea and main bronchi. This results from atrophy or absence of elastic fibers and smooth muscle within the airway wall, leading to loss of structural integrity and airway compliance [1]. Though suspected to be congenital in origin, MKS is often recognized later in life, with the average age of diagnosis in the third to sixth decade [2], typically due to its insidious onset and nonspecific respiratory symptoms [3].

Although first described histologically in 1897, MKS was clinically defined in 1932 and remains under‐recognized, with fewer than 500 cases reported in the literature to date [3, 4]. MKS has a striking male predominance ranging from 4:1 to 8:1 across different case series [3, 5]. A significant proportion of affected individuals are also chronic smokers [6], which may contribute to the progression of airway damage and symptomatology.

The pathophysiology of MKS includes not only tracheobronchomegaly but also the development of tracheal diverticulosis, impaired mucociliary clearance, and consequently increased risk for lower respiratory tract infections and bronchiectasis [3]. Bronchiectasis is reported in over 70% of patients with MKS, whereas tracheal diverticulosis occurs in approximately two‐thirds of cases [3]. These structural abnormalities contribute significantly to recurrent infections and chronic airway inflammation, which dominate the clinical presentation. In MKS, the symptoms are not very specific. A hallmark feature is chronic productive cough, often with purulent sputum, and occasional hemoptysis [7]. As tracheobronchial dilation progresses, the cough may become increasingly ineffective, contributing to secretion stagnation and recurrent bronchopulmonary infections. Patients may experience recurrent febrile episodes, and over time, develop exertional dyspnea and signs of chronic hypoxia such as central cyanosis and digital clubbing [7]. On physical examination, the only appreciable manifestation of MKS is coarse inspiratory crackles [7].

The precise etiology of MKS remains unclear. Although hypotheses suggest a congenital defect in the development of connective tissue in the tracheobronchial wall, this remains theoretical. MKS occurs sporadically, but rare familial clustering has been reported, raising the possibility of a genetic predisposition through an autosomal recessive mechanism [8]. MKS has also been associated with connective tissue disorders such as Ehlers–Danlos, Marfan′s syndrome, and cutis laxa; and rheumatologic disorders such as rheumatoid arthritis and ankylosing spondylitis [9, 10].

Diagnosis is typically made through imaging. Computed tomography (CT) is the modality of choice that reveals hallmark findings such as a tracheal diameter exceeding 25 mm in men and 21 mm in women (or > 27 mm sagittally), and bronchial dilatation [5]. Diagnosis can be difficult to establish using standard chest radiography, particularly on lateral views. In some cases, however, dilatation of the trachea and main bronchi may be visualized, with threshold diameters exceeding 3.0 cm for the trachea, 2.4 cm for the right main bronchus, and 2.3 cm for the left main bronchus. [11]. CT of the chest remains the gold standard for diagnosis, offering superior sensitivity in detecting tracheobronchial abnormalities. CT imaging also allows for comprehensive evaluation of the disease′s extent, including the identification of associated features such as tracheal diverticulosis and bronchiectasis.

Despite increased recognition, MKS is frequently misdiagnosed, with a reported median diagnostic delay of approximately 3 years [3]. Herein, we present a case of a middle‐aged male with known MKS who presented with pleuritic chest pain—a rarely emphasized complication. This case highlights the radiologic and clinical intricacies of MKS, while serving as a platform for reviewing its complications, diagnostic challenges and considerations, and current management strategies.

## 2. Case Presentation

A 56‐year‐old male with a history of bronchiectasis due to MKS (diagnosed over a decade ago), hypertension, mitral valve prolapse, and Type 2 diabetes mellitus presented to the emergency department with 3–4 days of intermittent, left‐sided pleuritic chest pain. He described the pain as sharp (rated 8/10), radiating to the posterior ribs, exacerbated by deep inspiration and leaning forward, and partially relieved when lying flat. The pain was nonexertional and persistent throughout the day. He denied any recent trauma to the chest.

The patient also reported a productive cough with yellow sputum, as well as chills, diaphoresis, night sweats, and occasional hot flashes. He denied fevers, recent sick contacts, upper respiratory symptoms, nausea, vomiting, reflux, lower extremity swelling, or gastrointestinal complaints. There was no history of recent travel, new medications, or illicit drug use. He is a lifelong nonsmoker and works as a forklift operator, with no recent physical strain.

He follows regularly with his pulmonologist and adheres to a pulmonary regimen including hypertonic saline nebulizations, fluticasone propionate/salmeterol, and albuterol. He uses his rescue inhaler 2–3 times daily with mild symptom relief and reported that morphine provided temporary pain relief during this episode. His most recent single‐photon emission computed tomography (SPECT) myocardial perfusion stress test in 2023 showed no evidence of inducible ischemia.

**Figure 1:**
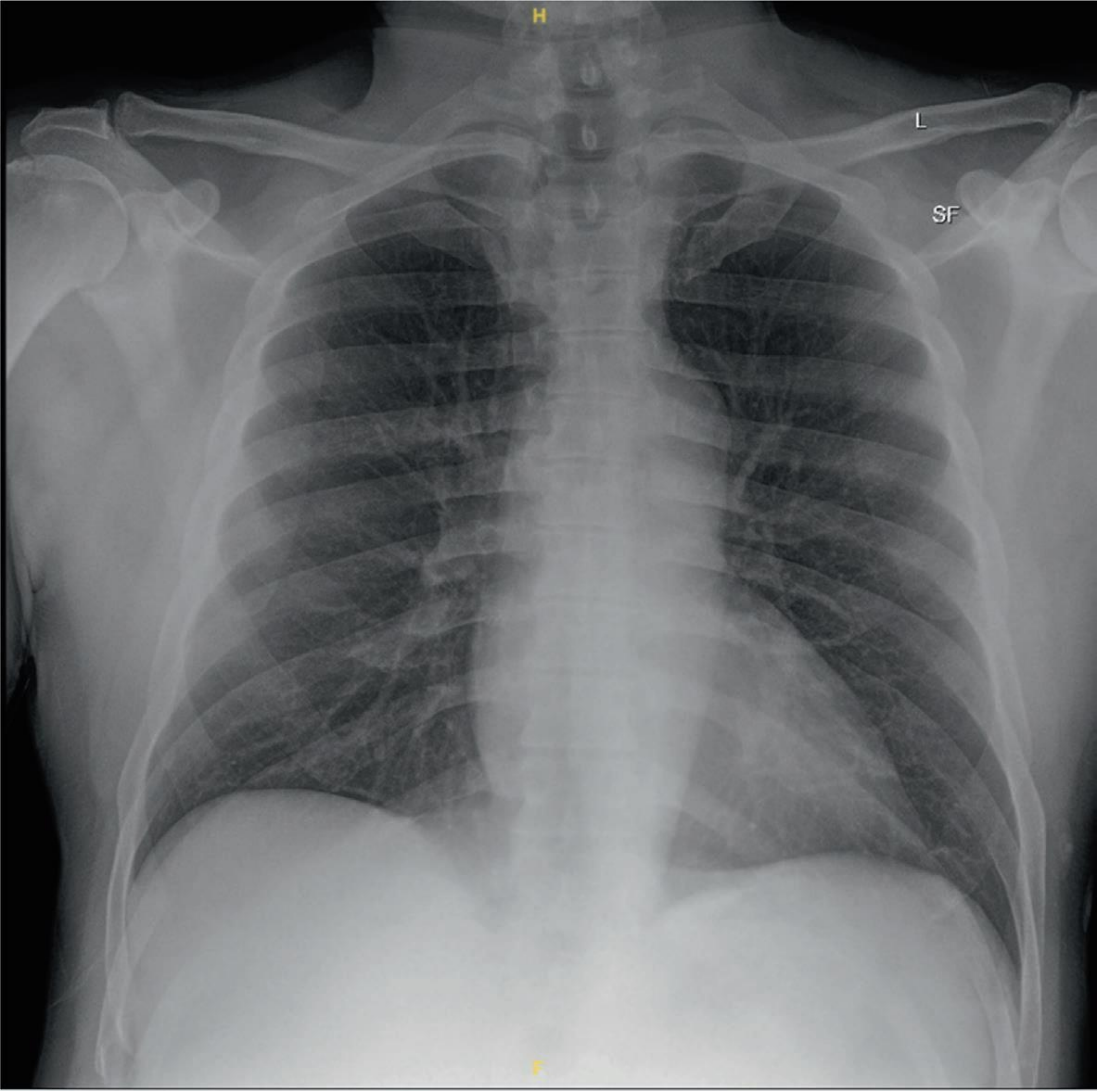
Chest radiograph on the day of admission showing no acute cardiopulmonary process. Lung volumes are preserved without evidence of consolidation, effusion, or pneumothorax.

**Figure 2:**
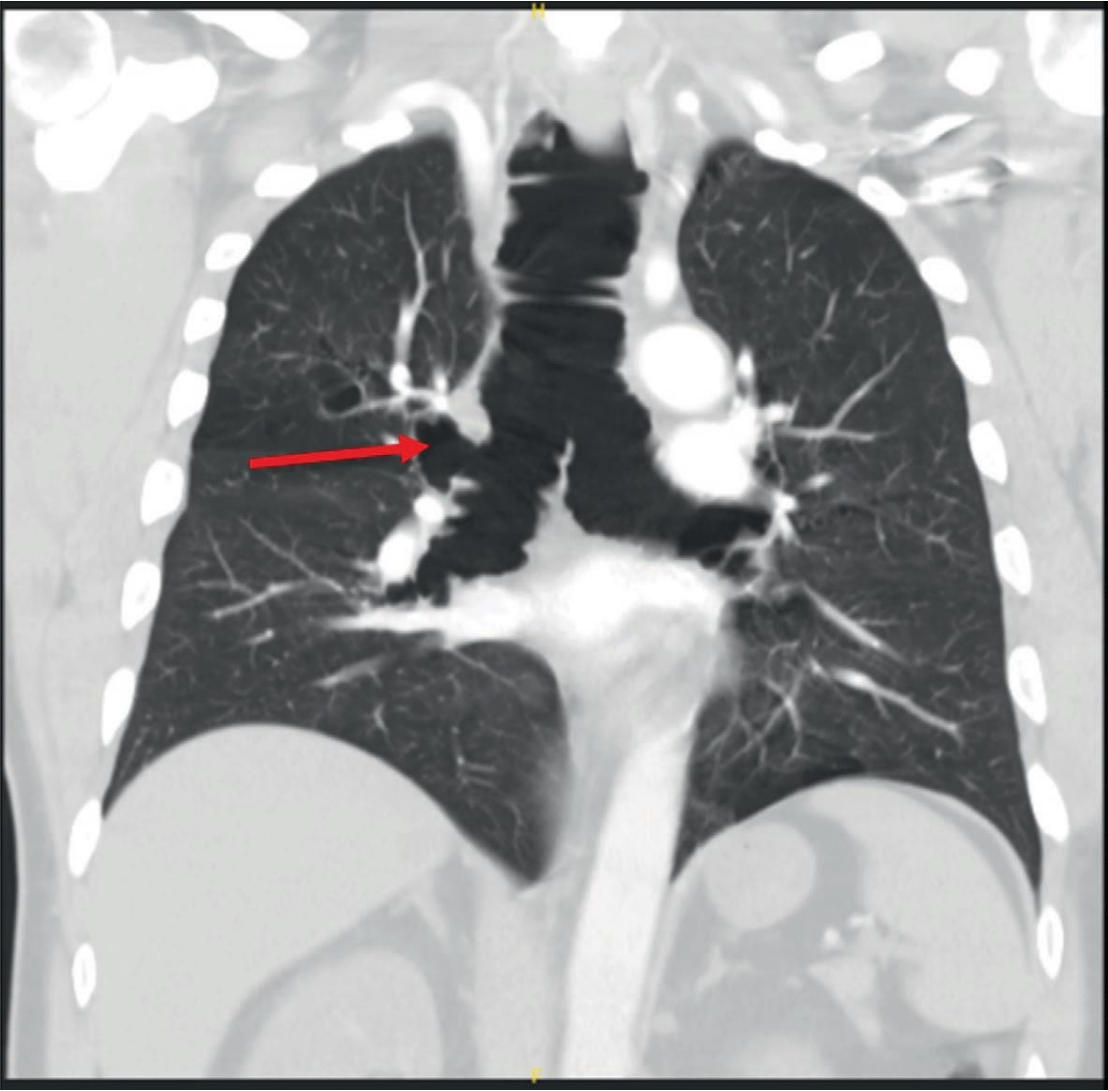
Coronal CT chest demonstrating marked tracheobronchomegaly, with tracheal and mainstem bronchial dilatation consistent with Mounier‐Kuhn syndrome. Red arrow highlights tracheal diverticulum.

**Figure 3:**
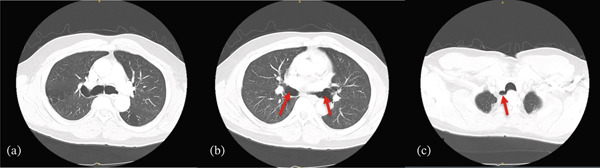
(a) Axial CT scan of the chest showing tracheal dilatation with anteroposterior and transverse widening of the trachea at the level of the carina. (b) Axial CT scan further highlighting the dilated main bronchi (red arrows), a characteristic feature of Mounier‐Kuhn syndrome. (c) Axial CT scan demonstrating a posterior right tracheal diverticulum (or outpouching), commonly associated with tracheobronchomegaly (red arrow).

In the emergency department, his blood pressure was elevated in the 180 s systolic. On physical exam, lung auscultation was clear bilaterally without wheezes or crackles; cardiac examination demonstrated a regular rate and rhythm without murmurs, and the chest wall was nontender to palpation. Laboratory evaluation revealed a creatinine of 1.28 mg/dL, GFR 66 mL/min/1.73m^2^, glucose 320 mg/dL, and normal sodium (135 mmol/L). Two sets of troponins were negative, and the electrocardiogram (ECG) showed normal sinus rhythm without acute ischemic changes. CBC was unremarkable, and the remainder of the metabolic panel was within normal limits. Rapid antigen testing for influenza A/B and SARS‐CoV‐2 was negative. Imaging included a chest x‐ray which showed no acute cardiopulmonary findings (Figure 1). A CTA of the chest ruled out pulmonary embolism and revealed stable chronic tracheobronchomegaly, a posterior right tracheal diverticulum, and right upper lobe cystic bronchiectasis, all consistent with known MKS (Figures 2 and 3). Quantitative airway measurements on coronal CT demonstrated a markedly enlarged trachea measuring 3.99 cm in transverse diameter, with right and left main bronchial diameters of 3.38 and 4.30 cm, respectively, consistent with tracheobronchomegaly. In the emergency department, he received multimodal analgesia including a one‐time dose of 500‐mg IV methocarbamol, and 4‐mg IV morphine, which was ordered every 4 h as needed for pain control. He was admitted to the hospital for further diagnostic workup, symptomatic management, and short‐term observation given the atypical nature of his pleuritic pain in the setting of underlying MKS.

Comprehensive immunologic workup was largely unrevealing: ESR and CRP were within normal limits, and alpha‐1 antitrypsin was low‐normal. Rheumatologic markers, including ANA, RF, p‐ANCA, c‐ANCA, proteinase 3, myeloperoxidase antibody, Jo‐1, Scl‐70, dsDNA, anticentromere, and chromatin antibodies, were all negative. Complement levels were within normal limits (C3: 128 mg/dL, C4: 37.9 mg/dL). The immunoglobulin panel showed mildly elevated IgG (1467 mg/dL), normal IgA (284.2 mg/dL), IgM (63 mg/dL), and low IgE (20.5 IU/mL). MRSA nares screen returned positive, and mupirocin was initiated bilaterally.

The patient was managed conservatively with the following medications:

• nebulized albuterol every 6 h
• nebulized budesonide twice daily
• nebulized 3% hypertonic saline nebulizers every 6 h
• guaifenesin twice daily
• daily proton pump inhibitor therapy (PPI)
• incentive spirometry
• flutter valve therapy
• gabapentin 100 mg twice daily for chest wall pain

He improved with supportive therapy and was discharged home with outpatient pulmonology follow‐up.

## 3. Discussion

MKS is defined by tracheal and main bronchial enlargement due to degeneration of the airway wall′s structural components. The pathophysiology involves loss of elastic and smooth muscle fibers, contributing to dynamic airway collapse, impaired secretion clearance, and chronic airway colonization [1]. These factors create a vicious cycle leading to bronchiectasis, tracheal diverticulosis, and progressive pulmonary decline.

There are three recognized subtypes of MKS: Type I involves slight symmetrical dilation of the trachea and/or the main bronchi, Type II features more pronounced dilatation with distinct diverticula, and Type III is marked by severe tracheobronchomegaly with extensive diverticula and sacculations extending to the distal bronchi [12]. Our patient has tracheobronchomegaly with a posterior tracheal diverticulum (Figure 3c) and right upper lobe cystic bronchiectasis, along with chronic stable airway dilation without severe distal airway involvement. His clinical course is characterized by chronic symptoms with intermittent exacerbations, but without severe respiratory failure, recurrent hospitalizations, or the need for advanced ventilatory support. These features are consistent with Type II (classic) MKS.

Although infectious or postinfectious sequelae are the most reported causes of symptom exacerbation in MKS, and the disease can remain asymptomatic in the absence of infection [13], our case uniquely featured pleuritic chest pain without infectious or thromboembolic findings, emphasizing an underappreciated dimension of MKS symptomatology. Although pleuritic chest pain in MKS is most attributed to infectious exacerbations, the precise mechanism underlying noninfectious pleuritic pain in MKS remains uncertain, and proposed explanations in such cases remain speculative. Given the absence of pleural abnormalities on imaging, the etiology in this case cannot be definitively established. The chest pain in our patient is postulated to be due to mechanical irritation of the pleura by inflamed adjacent airways or traction from structurally distorted bronchi. This highlights the need for greater clinical awareness of noninfectious exacerbations in MKS, particularly in patients presenting with pleuritic chest discomfort. The clinical improvement with pulmonary hygiene and bronchodilators further supports this interpretation.

Treatment of MKS remains largely supportive, focusing on airway clearance and prevention of complications. Current management strategies include vaccination, prompt antibiotic treatment for infectious exacerbations, mucolytics, inhaled hypertonic saline, and routine pulmonary physiotherapy to enhance secretion clearance [12]. Asymptomatic patients typically require no intervention, whereas symptomatic individuals benefit from aggressive pulmonary hygiene. Smoking cessation and avoidance of occupational irritants are strongly recommended to limit disease progression [3].

In severe cases, interventional therapies such as tracheal stenting and tracheobronchoplasty (TBP) have been employed with variable success. Surgery is generally limited due to the diffuse nature of airway involvement and lung transplantation has not demonstrated clear survival benefits [3]. In a prospective trial involving 12 patients with MKS, 11 of whom were men, TBP or airway stenting led to significant improvements in pulmonary function and health‐related quality of life [1]. Notably, 33% of these patients also had tracheal diverticula identified on bronchoscopy in addition to tracheobronchomegaly, reinforcing the high prevalence of diverticular involvement observed in MKS. Krustins et al. emphasize that management must consider the severity of airway collapse, comorbidities, and rate of disease progression, with multidisciplinary input from pulmonologists, radiologists, and thoracic surgeons [6].

Beyond bronchiectasis, reported complications include recurrent pneumothorax, hemoptysis, progressive respiratory insufficiency, and even rare associations with malignancies such as laryngeal or bronchogenic carcinoma [3, 6]. Additionally, tracheomalacia and expiratory airway collapse further compromise ventilation and can accelerate clinical decline [1, 6].

Therapeutic options remain limited and largely individualized due to the absence of randomized clinical trials. Conservative management remains the mainstay of care, and the patient described herein responded well to a regimen of bronchodilators, mucolytics, inhaled hypertonic saline, incentive spirometry, flutter valve therapy, and chest physiotherapy, stressing the value of supportive therapy in milder or moderate disease flares. This regimen is aimed at maintaining airway hygiene and reduce infectious burden [6]. Therapeutic options for MKS remain limited not only because the rarity of the syndrome makes large‐scale randomized trials infeasible but also because the underlying pathophysiology is incompletely understood. Significant variability in airway involvement, clinical trajectory, and structural severity further complicates the development of standardized treatment pathways.

Importantly, MKS progression is variable. Some patients maintain long‐term stability, whereas others experience rapid deterioration secondary to airway collapse or superimposed infections [6]. This variability highlights the importance of early diagnosis, structured follow‐up, and tailoring interventions to the individual′s clinical symptoms.

## 4. Conclusion

This case highlights the broad clinical variability of MKS, particularly its potential to manifest with atypical, noninfectious exacerbations such as pleuritic chest pain. Despite the absence of clear infectious triggers or thromboembolic disease, our patient experienced significant symptomatology attributable to the underlying structural airway abnormalities, stressing the need for clinicians to maintain a broad differential diagnosis when evaluating similar presentations. His favorable response to conservative, supportive therapy reinforces the central role of pulmonary hygiene and symptom‐directed management in MKS, particularly in the absence of severe respiratory compromise. Furthermore, this case emphasizes the importance of individualized, multidisciplinary care in optimizing patient outcomes, accounting for both the structural airway burden and dynamic clinical manifestations of the disease. As the current evidence base for MKS remains limited, especially concerning noninfectious presentations, this case adds to the growing recognition of pleuritic pain as a noteworthy, though underappreciated, component of MKS exacerbations. Future studies and longitudinal follow‐up are warranted to better delineate the full spectrum of complications and refine tailored management strategies for this rare airway disorder.

## Funding

No funding was received for this manuscript.

## Disclosure

This work was supported by HCA Healthcare and/or an HCA Healthcare affiliated entity. The views expressed in this publication represent those of the authors and do not necessarily represent the official views of HCA Healthcare or any of its affiliated entities.

## Consent

Patient consent was obtained.

## Conflicts of Interest

The authors declare no conflicts of interest.

## Data Availability

Data sharing is not applicable to this article as no datasets were generated or analyzed during the current study.
